# Associated factors with depression and sleep quality in T1DM patients: a cross-sectional descriptive study

**DOI:** 10.1186/s12888-023-04516-2

**Published:** 2023-01-09

**Authors:** Mi-Kyoung Cho, Mi Young Kim

**Affiliations:** 1 Department of Nursing Science, Chungbuk National University, Chungdae-Ro, Seowon-Gu, Cheongju, Korea; 2 College of Nursing, Hanyang University, 222 Wangsimni-Ro, Seongdong-Gu, Seoul, Korea

**Keywords:** Type 1 diabetes, Sleep quality, Depression, Resilience

## Abstract

**Background:**

Individuals with type 1 diabetes (T1DM) may experience sleep problems, usually due to low blood sugar levels during sleep or performance of blood sugar management (e.g., blood sugar monitoring). This study aimed to identify the disease-related characteristics, psychosocial aspects, and related factors underlying sleep quality in patients with T1DM.

**Methods:**

This study employed a descriptive research design. The participants were 159 individuals with T1DM who completed online questionnaires. The data were analyzed using descriptive statistics, correlations, and multiple regression analyses.

**Results:**

The average score for depression in T1DM patients was 23.77 (SD 5.31), and sleep quality received a score of 4.58 (SD 3.22). Depression was positively correlated with sleep quality and negatively correlated with the total resilience score. The factors linked to depression in T1DM patients were duration of disease, sleep latency, sleep duration, sleep disturbance, and resilience-acceptance of self and life sub-factors, with an explanatory power of 44.4% for the depression variance. The associated factors with sleep quality in T1DM patients were complications, resilience-personal competence sub-factors, and depression, with an explanatory power of 37.4% for sleep quality variance.

**Conclusions:**

The results of this study suggest that to improve sleep quality in patients with T1DM, it is necessary to develop and support disease management to prevent complications and implement interventions for improving resilience and reducing negative emotions such as depression.

## Introduction

Type 1 diabetes mellitus (T1DM) is an autoimmune condition resulting from the destruction of pancreatic beta cells [1]. T1DM is one of the most common chronic diseases in youth [2]. T1DM requires continuous adaptation and management, a complex and challenging process that includes diet management, exercise, insulin injections, and monitoring of blood glucose levels [3].

Individuals with T1DM, who usually undergo disease management throughout the rest of their lives, may experience psychosocial stress in managing their disease [4]. The incidence of psychosocial problems in children with T1DM was 55.9% higher than that in children without the disease. Specifically, irritation was the highest (38.1 %), followed by depression (36.9%) and anxiety (32.1%) [5]. Among the psychosocial problems experienced by individuals with T1DM, depression is one of the most common mental disorders [6]. While depression in individuals with T1DM can be associated with several factors, it has been associated with severe hypoglycemia, hospitalization due to complications, and poor quality of life [7].

For individuals with T1DM, managing psychosocial aspects, such as depression, is necessary. Although several studies have targeted children and adolescents due to the nature of T1DM, few studies in South Korea have targeted adults who need to manage the disease independently. T1DM is a lifelong disease, and its management requires treatment givers to pay attention to depression in adults with social lives. Therefore, it is necessary to (1) investigate depression levels in individuals with T1DM, as this is a representative psychological aspect, and (2) identify the related factors of this psychological aspect.

Individuals with T1DM face another easily-overlooked but important physical problem–sleep quality. Sleep is essential for activating bodily functions and maintaining good health. Poor sleep quality can cause fatigue, poor memory and concentration, restlessness, and anxiety, thus affecting the quality of life [8]. Furthermore, sleep can significantly affect overall health. Sleep deprivation in adults can increase the risks of developing cardiovascular disease, diabetes, obesity, cancer, hypertension, dyslipidemia, and physical stress; impair the immune system and coping abilities; directly or indirectly affect mortality [9]. In a study targeting general adults, sleep quality was low when depression was high [10]. Moreover, a study on adolescents found that sleep deprivation is more likely to lead to the development of a major depressive disorder [11]. Thus, sleep is associated with health and is closely related to health, even in adults.

Along with research on healthy adults, research has also examined the sleep quality of those suffering from diseases that can cause sleep disturbances; these include patients undergoing hemodialysis and those receiving treatment for stroke and asthma [12–14]. Research on T1DM has reported hyperglycemia, hypoglycemia, sleep disturbances caused by glucose fluctuations, and sleep problems [15, 16]. Poor sleep quality has also been reported in children and adolescents with T1DM [17, 18]. Sleep deprivation has been reported in nearly half of examined children and up to 77% of examined adolescents with T1DM [19–21]. Sleep plays an important role in managing the disease, as sleep disturbances and nighttime sleep duration or quality fluctuations are associated with inadequate lifelong management of T1DM [22, 23]. T1DM management is closely related to sleep, as higher sleep variability influences poor blood sugar control in individuals with T1DM [24]. Specifically, it is important to focus on and intervene in the sleep quality of adults with T1DM, as early intervention may be necessary for successfully managing the condition of adults who actively participate in socioeconomic activities [9]. Resilience in people with diabetes has been described as the ability to achieve health and psychosocial outcomes, despite the numerous difficulties associated with living with and managing diabetes [25]. Resilience is a positive asset that can offset negative aspects (e.g., depression and sleep disturbances) related to T1DM disease management. Resilience can be considered a factor affecting depression and sleep in individuals with T1DM, as it is a positive adaptive response to stressful situations and represents a mechanism for overcoming difficult experiences [26]. It has been reported that resilience in adolescents with T1DM reduces negative emotions associated with diabetes, increases self-efficacy behaviors in diabetes management, and promotes metabolic regulation [27]. Therefore, this study aimed to examine the effects of general and disease-related characteristics, including resilience, on depression and sleep in adults with T1DM. Furthermore, we aimed to provide basic data for the development of depression- and sleep-related interventions for individuals with T1DM.

## Materials and methods

This cross-sectional descriptive study aimed to identify the associated factors with depression and sleep quality in T1DM patients.

### Participants

The study participants were patients with T1DM who were enrolled as members of a T1DM online community. The participant selection criteria were as follows: diagnosed with T1DM for more than one year, managed diabetes at home, and could voluntarily access the survey URL to read and respond to the survey. Furthermore, the participant exclusion criteria were as follows: any mental or neurological disease, any participation in any depression and sleep quality improvement programs, and any hospitalization due to complications during the data collection period.

The optimal study sample size was calculated using the sample size calculation program G Power 3.1.9.4 [28] to determine the minimum sample size for performing multiple linear regression with the following parameters: ten characteristic variables of participants as independent variables (age, sex, religion, job, duration of disease, complications, education of diabetes, experience of diabetes camp, source of diabetes information, and HbA1c). Two variables of either sleep quality or depression and resilience, as well as a two-sided significance level (α) of 0.05, a statistical power (1-β) of 0.90, and a median effect size (f2) of 0.15 [29]. A total of 157 participants were required. Efforts were made to avoid a potential selection bias during participant recruitment. Finally, 159 participants participated in this study.

### Assessments

#### Characteristics of the Participants

Age, sex, religion, job, duration of disease, presence or absence of T1DM complications (macrovascular and microvascular), education about diabetes, the experience of diabetes camp, source of diabetes information, and HbA1c values (recently measured within the last three months) were the characteristics of the study participants. Age and duration of disease were calculated by subtracting the year of birth and the diagnosis year, respectively, from the year of data collection; for HbA1c (%), only the value was provided in numbers. Sex, a categorical variable, required a response of “male” or “female,” and religion, job, presence or absence of T1DM complications, education about diabetes, and experience of diabetes camp required a response of “yes” or “no.” The source of diabetes information was checked as “personal” if it was found through the Internet, books, or in person, and “expert” if it was received through education from experts (e.g., doctors or nurses in hospitals or public health centers).

#### Depression

Depression is a common and serious medical illness that negatively affects how individuals feel, think, and act [30]. Depression was measured using the Center for Epidemiologic Studies Depression Scale (CES-D) developed by Radloff to evaluate depressive symptoms [31]. Cho and Kim validated this scale in Korean, and its items measured depression symptoms experienced during the past seven days [32]. The CES-D includes 20 items that evaluate the following depressive symptoms: depressive emotions, positive emotions, interpersonal relationships, and physical deterioration. The items were ranged as follows on a 4-point Likert scale: “Rarely or None of the Time (Less than 1 Day)” = 1 point, “Some or a Little of the Time (1-2 Days)” = 2 points, “Occasionally or a Moderate Amount of Time (3-4) Days)” = 3 points, and “Most or All of the Time (5-7 Days)” = 4 points. Among these, positive items (5, 10, and 15) were converted in reverse when calculating the scores. The total score ranged from 0 to 60, with higher scores indicating more severe depressive symptoms. The tool’s reliability was determined to be as follows at various times: a Cronbach’s α of .85 for the Radloff tool [31], a Cronbach’s α of .89~.93 for the Korean version by Cho and Kim [32], and .764 in this study.

#### Sleep Quality

Sleep quality was defined as an individual’s satisfaction with personal sleep experience; this factor incorporates aspects of sleep initiation, sleep retention, sleep quantity, and freshness in wakefulness [33]. Sleep quality was measured using the Pittsburgh Sleep Quality Index (PSQI) and validated in Korea by Choi et al. [34, 35]. This scale is a self-report questionnaire that measures the past month’s sleep quality and degree of discomfort while sleeping. The four questions dealt with the time of going to bed, time taken to fall asleep, time of waking up, and duration of actual sleep. Furthermore, other questions dealt with various factors occurring during sleep, frequency of taking sleeping pills, and frequency of work interruption. The tool scored 0 points for “not during the past month,” 1 point for “less than once a week,” 2 points for “once or twice a week,” and 3 points for “three or more times a week.” The final question on perceptual sleep quality was scored on a scale as follows: 0 points for “very good,” 1 point “for fairly good,” 2 points for “fairly bad,” and 3 points for “very bad.” These raw scores were divided into seven components (sleep quality, sleep latency, sleep duration, habitual sleep efficiency, sleep disturbance, use of sleeping medication, and daytime dysfunction); each component scored from 0 to 3 points. The sum of the seven components is called the global PSQI score, which ranges from 0 points (no sleep problems) to 21 points (severe sleep disturbances). Based on a global PSQI score of 5, a score of less than 5 indicates that the scorer is a good sleeper and a score of 5 or more indicates that the scorer is a poor sleeper. The tool’s reliability was as follows at various times: a Cronbach’s α of .83 at the time of its development [34], a Cronbach’s α of .782 for the Korean version [35], and a Cronbach’s α of .777 for the current study.

#### Resilience

Resilience is the process of adapting well to adversity, trauma, tragedy, threats, or serious stressors [36]. The resilience scale was developed by Wagnild and Young and developed in the Korean version [37]. The tool consisted of 25 items, including 17 on personal competence and 8 on acceptance of self and life; these factors formed two sub-factors. Scores were measured on a 7-point Likert scale (range: 1 point for “disagree” to 7 points for “agree”). The total score ranged from 25 to 175, with higher scores indicating higher resilience, below 121 indicating low resilience, between 121 and 145 indicating moderate resilience, and a score exceeding 145 indicating moderately high to high resilience. Regarding reliability, a Cronbach’s alpha of .85 was reported by Wagnild and Young at the time of development [37]; the current study reported a Cronbach’s alpha of .942.

### Ethical considerations

The study participants were informed about the study purpose and procedures, their participation-related rights, and their guaranteed anonymity. Furthermore, only individuals who read the online consent form that included a study description and voluntarily consented to participate could participate in the survey.

### Setting and data collection

The data was collected from one online community (https://cafe.naver.com/dmtype1) of T1DM patients in Korea. The participants visited the community website, read the participant recruitment instructions, and voluntarily accessed the survey URL through mobile or web to participate in the study. Only T1DM patients could join this online community. When signing up for an online community, the patient’s real name, date of birth, phone number, e-mail address, home address, and treatment hospital must be entered as personal information.

Data were collected through an online survey from August 3 to 14, 2020. The researchers explained the study purpose and procedures to the managers of the T1DM online communities and asked them to cooperate and get consent from T1DM patients. Participant recruitment-related information (purpose, procedure, participation method, rewards, etc.) regarding the online survey was posted on the T1DM online community website. To give coupons to patients as a reward, a phone number was written on the questionnaire, and after data collection, the cafe keeper was asked to check whether the patient was a member of the cafe. Data were collected from 159 T1DM patients.

### Bias and sensitivity analysis

As this study was cross-sectional, it was difficult to identify and measure potential confounding factors in the cohort design. Nevertheless, in this study, to control for potential confounding variables, variables that can affect the outcome variables and the characteristics of participants were identified, and multiple regression analysis was conducted to confirm whether the confounding variables affected the outcome variables. In the regression analysis, only significant explanatory variables in the univariate analysis were entered into the model. According to the F probability distribution, a variable was entered into the model when the significance level of the F value was less than .05 and was removed when the significance level was greater than .10. The cut-off for statistical significance in the present study was *p* <.05.

### Statistical analysis

The collected data were analyzed using the SPSS Statistics software (version 25.0; SPSS, IBM, New York, USA). One missing data in the duration of the disease was treated as a mean imputation. The participant characteristics were analyzed using frequency, percentage, mean, and standard deviation. Depression, sleep quality, and resilience were analyzed using mean, standard deviation, and range through descriptive statistics. The differences in depression and sleep quality, which were outcome variables according to participant characteristics, were analyzed using an independent t-test and one-way ANOVA. A post-hoc analysis was performed using Scheffe’s test. Correlations among sleep quality, resilience, and depression in T1DM patients were analyzed using Pearson’s correlation. Associated factors with depression and sleep quality in T1DM patients were identified using stepwise multiple regression.

## Results

### Characteristics of the participants

The study participants comprised 159 T1DM patients aged 32.29 (SD 9.89) years, including 108 (67.9%) females; out of the total recruited participants, 127 (79.9%) were working, and 114 (71.7%) were non-religious. The average duration of the disease was 12.88 (SD 8.23) years, and 37 (23.3%) complications were reported. The average HbA1c level measured recently was 7.21 (SD 1.27%), and 46 participants (28.9%) had an HbA1c level of 8.0% or higher. The average duration of the disease was 12.88 (SD 8.23) years, and 37 (23.3%) complications were reported. While 144 (90.6%) participants received education on diabetes, 42 (26.4%) had the experience of diabetes camp. The sources of diabetes information were “personal” for 121 (76.1%) (e.g., Internet, books, diabetes communities, and acquaintances). The average resilience score was 122.80 (SD = 23.78), and 64 (40.3%) participants had low-resilience scores (Table 1).

**Table 1:** Characteristics of the participants (*n* = 159)

Characteristics		N (%)	Mean ± SD
Age (year)	< 29	69 (43.4)	32.29 ± 9.89
	30 ~ 49	83 (52.2)	
	≥ 50	7 (4.4)	
Sex	Female	108 (67.9)	
	Male	51 (32.1)	
Religion	No	114 (71.7)	
	Yes	45 (28.3)	
Job	No	32 (20.1)	
	Yes	127 (79.9)	
Duration of disease (year)	< 10	66 (41.5)	12.88 ± 8.23
	≥ 10	93 (58.5)	
Complication	No	122 (76.7)	
	Yes	37 (23.3)	
Education of diabetes	No	15 (9.4)	
	Yes	144 (90.6)	
Experience of diabetes camp	No	117 (73.6)	
	Yes	42 (26.4)	
Source of diabetes information	Personal	121 (76.1)	
	Expert	38 (23.9)	
HbA1c (%)	< 6.5	50 (31.4)	7.21 ± 1.27
	6.5 ~ 7.9	63 (39.6)	
	≥ 8.0	46 (28.9)	
Resilience	Low	64 (40.3)	122.80 ± 23.78
	Moderate	65 (40.9)	
	High	30 (18.9)	

*SD *standardized deviation, *HbA1c* Glycated hemoglobin

### Descriptive statistics of variables

The average score for depression in T1DM patients was 23.77 (SD 5.31), and sleep quality received a score of 4.58 (SD 3.22). Among the components of sleep quality, sleep latency, daytime dysfunction, sleep quality, and sleep disturbance had the highest scores, and sleeping medication received the lowest score (Table 2).

**Table 2:** Descriptive statistics of the variables (*n* = 159)

Variables	Sum of score		Converted score	
	Mean ± SD	Range	Mean ± SD	Range
Depression	23.77 ± 5.31	0 ~ 60	1.19 ± 0.27	0 ~ 3
Sleep quality	4.58 ± 3.22	0 ~ 15		
Sleep quality	1.37 ± 0.80	0 ~ 3		
Sleep latency	1.51 ± 1.04	0 ~ 3		
Sleep duration	0.89 ± 0.99	0 ~ 3		
Habitual sleep efficiency	0.47 ± 0.90	0 ~ 3		
Sleep disturbance	1.32 ± 0.62	0 ~ 3		
Use of sleeping medication	0.36 ± 0.89	0 ~ 3		
Daytime dysfunction	1.43 ± 0.98	0 ~ 3		

*SD *standardized deviation

### Differences in depression and sleep quality based on characteristics of the participants

According to the participants’ characteristics, participants without religion and those without experience of diabetes camp had a higher degree of depression. Depression was higher in the low-resilience group compared to the moderate- and high-resilience groups. Sleep quality was significantly higher in participants who were non-religious, had no experience of diabetes camp, had a longer duration of the disease, and had complications. It was also significantly higher in the low-resilience group compared to the moderate- and high-resilience groups. According to other participant characteristics, there was no difference in the degree of depression or sleep quality (Table 3).

**Table 3:** Depression and sleep quality by characteristics of the participants (*n* = 159)

Characteristics		Depression		Sleep quality	
		Mean ± SD	*t or F (p)* *Scheffe*	Mean ± SD	*t or F (p)* *Scheffe*
Age (year)	< 29	24.09 ± 4.65	0.47 (0.626)	4.77 ± 2.97	0.68 (0.506)
	30 ~ 49	23.65 ± 5.73		4.34 ± 3.37	
	≥ 50	22.14 ± 6.67		5.57 ± 3.87	
Sex	Female	23.95 ± 5.31	0.62 (0.535)	4.62 ± 3.25	0.24 (0.813)
	Male	23.39 ± 5.34		4.49 ± 3.17	
Religion	No	24.38 ± 5.47	2.31 (0.022)	5.04 ± 3.32	3.30 (0.001)
	Yes	22.24 ± 4.58		3.40 ± 2.61	
Job	No	24.22 ± 5.66	0.53 (0.597)	4.72 ± 3.27	0.27 (0.784)
	Yes	23.66 ± 5.23		4.54 ± 3.22	
Duration of disease (year)	< 10	23.62 ± 5.84	-0.48 (0.634)	5.00 ± 3.44	2.00 (0.047)
	≥ 10	24.03 ± 4.54		4.00 ± 2.83	
Complication	No	23.70 ± 5.07	-0.30 (0.768)	4.24 ± 2.89	-2.09 (0.042)
	Yes	24.00 ± 6.10		5.70 ± 3.95	
Education of diabetes	No	26.27 ± 3.61	1.93 (0.056)	4.40 ± 2.20	-0.23 (0.822)
	Yes	23.51 ± 5.40		4.60 ± 3.31	
Experience of diabetes camp	No	24.27 ± 5.53	2.22 (0.029)	4.89 ± 3.28	2.05 (0.042)
	Yes	22.38 ± 4.41		3.71 ± 2.91	
Source of diabetes information	Personal	23.93 ± 5.30	0.64 (0.521)	4.72 ± 3.23	0.98 (0.328)
	Expert	23.29 ± 5.36		4.13 ± 3.16	
HbA1c (%)	< 6.5	23.58 ± 5.56	1.55 (0.215)	4.70 ± 3.39	2.18 (0.117)
	6.5 ~ 7.9	23.11 ± 5.00		3.98 ± 2.74	
	≥ 8.0	24.89 ± 5.38		5.26 ± 3.53	
Resilience	Low^a^	26.16 ± 5.38	13.12 (< 0.001)	5.50 ± 3.04	6.97 (0.001)
	Moderate^b^	22.57 ± 5.27	a > b, c	4.42 ± 3.50	a > b, c
	High^c^	21.30 ± 2.69		2.97 ± 2.14	

*CES-D* Center for Epidemiologic Studies Depression Scale, *PSQI* Pittsburgh Sleep Quality Index, *SD *standardized deviation*, HbA1c* Glycated hemoglobin

Superscript a, b, c post-hoc Scheffe test factors which are compared to a reference category

### Correlation of depression and other variables

For all participants, depression was positively correlated with sleep quality and negatively correlated with the total resilience score: resilience-personal competence and resilience-acceptance of self and life. Furthermore, sleep quality and total resilience scores were negatively correlated: resilience-personal competence and resilience-acceptance of self and life (Table 4).

**Table 4:** Correlations of the variables (*n* = 159)

Variables	Sub-factors	Depression	Sleep quality
		*r (p)*	
Depression		1	
Sleep quality		0.58 (< 0.001)	1
Resilience	Personal competence	-0.39 (< 0.001)	-0.37 (< 0.001)
	Acceptance of self and life	-0.40 (< 0.001)	-0.36 (< 0.001)
	Total score	-0.41 (< 0.001)	-0.38 (< 0.001)

### Associated factors with depression and sleep quality

To identify liked factors to depression and sleep quality in T1DM patients, a stepwise multiple regression analysis was conducted with age, duration of disease, serum HbA1c, and the sub-factors of resilience that can be associated with depression and sleep quality as continuous variables. Categorical variables of sex, religion, job, complication, education about diabetes, the experience of diabetes camp, and source of diabetes information were used as dummy variables. The assumptions of multiple regression analysis, such as normal distribution, equal variance, and linearity, were satisfied, and there were no multicollinearity problems among the independent variables (Table 5).

**Table 5:** Associated factors with depression and sleep quality (*n* = 159)

Variables	Depression			Sleep quality		
	*B*	*SE*	*t (p)*	*B*	*SE*	*t (p)*
Intercept	22.12	2.21	10.03 (< 0.001)	-0.53	1.82	-0.29 (< 0.001)
Duration of disease	-0.08	0.04	-1.98 (0.049)			
Complication				1.25	0.49	2.56 (0.011)
Sleep latency	0.96	0.37	2.58 (0.011)			
Sleep duration	1.03	0.36	2.83 (0.005)			
Sleep disturbance	3.35	0.59	5.65 (< 0.001)			
Resilience-acceptance of self and life	-0.11	0.04	-2.45 (0.016)			
Resilience-personal competence				-0.03	0.01	-2.22 (0.028)
Depression				0.31	0.04	7.47 (< 0.001)
* F (p)*	25.47 (< 0.001)			32.21 (< 0.001)		
Adj. R^2^	44.4			37.4		
Tolerance	0.71 ~ 0.99			0.84 ~ 0.98		
VIF	1.00 ~ 1.41			1.02 ~ 1.20		
Durbin-Watson	2.09			1.92		

*B* Unstandardized Regression Coefficient*, SE* standard error*, Adj. R*^2^ adjusted coefficient of determination, *VIF* Variance Inflation Factor

The regression models were analyzed using the stepwise method of multiple linear regression

In the depression model, the factors that liked to depression in T1DM patients were duration of disease, sleep latency, sleep duration, sleep disturbance, and resilience-acceptance of self and life sub-factors, with an explanatory power of 44.4% for the depression variance. The longer the duration of the disease, sleep latency, and sleep duration, the higher the sleep disturbance among the components of sleep quality; the lower the resilience-acceptance of self and life sub-factors, the higher the degree of depression. The factors associated with sleep quality in T1DM patients were complications, resilience-personal competence sub-factors, and depression, with an explanatory power of 37.4% for sleep quality variance. In the sleep quality model, a greater number of complications was associated with a lower degree of resilience-personal competence sub-factors; furthermore, greater severity of depression was associated with lower sleep quality.

## Discussion

This study investigated the associated factors with depression and sleep in individuals with T1DM. The results were as follows.

In the depression model, a shorter duration of disease was associated with a longer sleep latency, longer sleep duration was associated with a higher degree of sleep disturbance, and a lower level of resilience-acceptance of self and life sub-factors related to a higher degree of depression. The model with these variables had an explanatory power of 44.4%. This study found that sleep quality was negatively associated with depression. This finding is consistent with previous studies involving the general public and individuals with T1DM, which found that poor sleep quality aggravated depressive symptoms [38, 39]. Poor sleep quality is associated not only with depression but also with glucose levels, hyperglycemia, behavioral disorders, poor quality of life, poor grades, depression, sleep-wake behavior problems, and drowsiness [39]. Individuals with T1DM may have more sleep-disturbing factors than the general population; this suggests that it may negatively affect adults who have to be productive at work. This study found that lower resilience-acceptance of self and life sub-factors was associated with a higher level of depression.

Resilience, as construed by Wagnild, comprises five essential characteristics of a meaningful life (purpose): perseverance, self-reliance, equanimity, and existential aloneness (i.e., coming home to yourself) [40]. Among the sub-factors, “acceptance of self and life” is an aspect of accepting and accommodating diabetes, as captured in the items “I usually take things in stride” and “My life has the meaning” [41]. These are related to psychosocial aspects such as depression. Resilience represents a complex set of protection and health-triggering factors whose processes are important for understanding health and disease, treatment, and healing. As it is a protective factor that induces individuals to grow more resilient to adverse events, thus leading to positive developmental outcomes, the lack of this protective factor is thought to affect depression in individuals with T1DM [26]. This finding supports a previous study on the mediating role of resilience in the relationship between negative life events and depression [42]. Therefore, as individuals with T1DM are not well-managed in terms of depression, intervention programs are necessary for alleviating their negative emotions, considering that depression is highly correlated with T1DM self-management [43].

In the sleep quality model, a greater number of complications were associated with a lower level of resilience-personal competence sub-factors, and a greater degree of severity in depression related to lower sleep quality. The model had an explanatory power of 37.4%. Sleep is essential for activating physical functions and maintaining health. Lack of sleep can negatively affect an individual’s quality of life by causing fatigue, poor memory and concentration, restlessness, and anxiety [8]. It plays an important role in healthy adults; however, the effects of poor sleep quality can be even more damaging in adults with diseases. As a result of this study, there was no statistically significant difference in the degree of depression according to sex. According to previous studies, women were more likely to belong to the group experiencing depressive symptoms than men [44]. On the other hand, a study on type 1 diabetes reported no difference in depression according to gender [45]. Further studies on gender-specific depression in T1DM are needed.

This study’s results showed that a greater number of complications was associated with lower sleep quality, consistent with a previous study, which reported that a poorer health level predicted lower sleep quality among adults overall [46]. The results also showed that resilience affected sleep, as measured by the resilience-acceptance of self and life and resilience-personal competence subscales [37]. Personal competence seems to be associated with the practical management of diabetes, as indicated by the items “Keeping an interest in things is important” and “I have self-discipline” [41]. This result is consistent with that of a previous study that reported that good resilience prevents the onset and exacerbation of the disease, promotes good health, promotes recovery, and provides a productive life and sense of well-being despite chronic illness [26]. Therefore, strengthening resilience can contribute to health promotion, and in the case of diseases, it can alleviate them and accelerate and promote healing. Therefore, it is necessary to introduce programs to strengthen resilience. This study also found that a severe degree of depression was linked to lower sleep quality, thus indicating a relationship between depression and sleep. This result is consistent with that of a previous study, which found that a higher level of depression predicted lower sleep quality in adults overall [10]. These sleep problems can be particularly problematic for individuals with T1DM, as they can be negatively linked to insulin sensitivity, disease progression, and the development of complications [15]. A systematic review of sleep and diabetic management in individuals with T1DM suggested that lower sleep quality (e.g., excessive or insufficient sleep, sleep disturbance, and sleep variability) was associated with inadequate T1DM management [47]. Therefore, it is necessary to include sleep education in managing T1DM.

Among the associated factors with sleep quality, only complications were associated with disease-related factors, while the duration of disease and HbA1c level were not related factors. This result is inconsistent with that of a previous study that indicated a relationship between HbA1c levels and sleep quality. Although the direct relationship with HbA1c levels is unclear from this finding and previous studies that have considered the relationship between psychosocial aspects and sleep, it seems that the psychological impact of diabetes management is significant. The inconveniences and stress caused by nocturnal blood glucose monitoring, fear of hypoglycemia during sleep, and treatments and psychosocial stress associated with T1DM support the finding that individuals with T1DM tend to have difficulty initiating and maintaining sleep [47]. Managing T1DM can be considered to involve controlling blood sugar, but this study showed that HbA1c was not directly related to sleep quality, thus suggesting that managing psychosocial aspects, such as depression and resilience, is necessary. Adults with T1DM may have a social life, jobs, and productive work; however, if their sleep quality is low, they may be unable to expend energy on their daytime activities efficiently due to fatigue. Since T1DM requires lifetime management, enabling patients to enjoy a productive social life even while suffering from the disease is important for increasing their quality of life and satisfaction. Furthermore, the current study reported that resilience is associated with sleep and depression. Strengthening resilience is necessary, as it plays an important role in promoting and protecting mental health against several risk factors (e.g., stressful life events) and enhancing the ability to cope with crises by reinforcing protective factors such as active coping [48].

### Study limitations

The limitations of this study were as follows. Since this study was cross-sectional, the regression analysis results did not imply a causal relationship between the independent variable and the dependent variable as a factor. The regression model in this study was a descriptive model that attempts to explain the linearity of the independent variable to the dependent variable. Therefore, researchers should focus on investigating the relationship between these variables as a causal relationship. This study included HbA1c as a characteristic indicator for blood sugar control; however, it is necessary to include not only HbA1c as an index related to sleep or depression but also blood sugar variance or degree of hypoglycemia as a related variable. Therefore, various other factors that can be linked to depression and sleep should be considered in future studies. Furthermore, combining the methods to objectively measure sleep quality is necessary. There are limitations in generalizing the results of this study.

## Conclusions

An examination of the related factors with depression and sleep in adults with T1DM showed that duration of disease, sleep, and resilience was associated with depression, while complications, depression, and resilience were associated with sleep. Resilience was a positive factor in alleviating the negative effects of T1DM. The psychosocial and sleep-related characteristics of individuals with T1DM identified in this study could be used for developing self-management guidelines in clinical practice. Furthermore, it is expected that assessment and efficient management of the psychosocial and sleep problems experienced by individuals with T1DM will improve their quality of life by enhancing their physical and emotional states.

Based on the current study results, we offer the following suggestions. First, following the results of this study, a long-term longitudinal prospective follow-up study is suggested to assess the degree of the relevant health problems (e.g., sleep disturbances and depression) in individuals with T1DM. Second, follow-up studies are suggested to develop a nursing intervention that can strengthen resilience—a variable that affects depression and sleep quality in individuals with T1DM—and verify the suggested program’s effectiveness.

## Data Availability

The datasets generated and/or analyzed during the current study are not publicly available due to privacy regulations but are available from the corresponding author at a reasonable request.

## Abbreviations

CES-D: Center for Epidemiologic Studies Depression Scale
PSQI: Pittsburgh Sleep Quality Index
SD: Standardized deviation
HbA1c: Glycated hemoglobin

## References

[1] Perfect MM (2020). Sleep-related disorders in patients with type 1 diabetes mellitus: current insights. Nat Sci Sleep.

[2] Mayer-Davis EJ, Lawrence JM, Dabelea D, Divers J, Isom S, Dolan L (2017). Incidence Trends of Type 1 and Type 2 diabetes among Youths, 2002–2012. N Engl J Med.

[3] Kumar KM, Saboo B, Rao PV, Sarda A, Viswanathan V, Kalra S (2015). Type 1 diabetes: Awareness, management, and challenges: current scenario in India. Indian J Endocrinol Metab.

[4] Tareen RS, Tareen K (2017). Psychosocial aspects of diabetes management: dilemma of diabetes distress. Transl Pediatr.

[5] Khandelwal S, Sengar GS, Sharma M, Choudhary S, Nagaraj N (2016). Psychosocial illness in children with Type 1 diabetes mellitus: prevalence, pattern and risk factors. J Clin Diagn Res.

[6] Al-Khurinej A (2007). Emotional and behavioral problems among diabetic children. Dig Middle East Stud.

[7] Kristensen LJ, Birkebaek NH, Mose AH, Hohwü L, Thastum M (2014). Symptoms of emotional, behavioral, and social difficulties in the Danish population of children and adolescents with type 1 diabetes–results of a national survey. PLoS One.

[8] Edéll-Gustafsson UM, Kritz EI, Bogren IK (2002). Self-reported sleep quality, strain, and health in relation to perceived working conditions in females. Scand J Caring Sci.

[9] Luca G, Haba Rubio J, Andries D, Tobback N, Vollenweider P, Waeber G (2015). Age and gender variations of sleep in subjects without sleep disorders. Ann Med.

[10] Strine TW, Chapman DP (2005). Associations of frequent sleep insufficiency with health-related quality of life and health behaviors. Sleep Med.

[11] Roberts RE, Duong HT (2014). The prospective association between sleep deprivation and depression among adolescents. Sleep.

[12] Han O, Park HJ (2020). A study on the correlation of sexual function, depression, and sleep quality in hemodialysis male patients. J Korea Academia-Industrial Cooperation Society.

[13] Ju SH, Kim H (2017). Impact of sleep quality and pain degree on the activities of daily living in patients with stroke. J Korean Soc Occup Ther.

[14] Chung MH, Park H (2016). Symptom experiences, sleep quality and quality of life for patients with asthma. J Korea Academia-Industrial Cooperation Society.

[15] Farabi SS (2016). Type 1 diabetes and sleep. Diabetes Spectr.

[16] van Dijk M, Donga E, van Dijk JG, Lammers GJ, van Kralingen KW, Dekkers OM (2011). Disturbed subjective sleep characteristics in adult patients with long-standing type 1 diabetes mellitus. Diabetologia.

[17] Pillar G, Schuscheim G, Weiss R, Malhotra A, McCowen KC, Shlitner A, Peled N, Shehadeh N (2003). Interactions between hypoglycemia and sleep architecture in children with type 1 diabetes mellitus. J Pediatr.

[18] Villa MP, Multari G, Montesano M, Pagani J, Cervoni M, Midulla F, Cerone E, Ronchetti R (2000). Sleep apnoea in children with diabetes mellitus: effect of glycaemic control. Diabetologia.

[19] Estrada CL, Danielson KK, Drum ML, Lipton RB (2012). Insufficient sleep in young patients with diabetes and their families. Biol Res Nurs.

[20] McDonough RJ, Clements MA, DeLurgio SA, Patton SR (2017). Sleep duration and its impact on adherence in adolescents with type 1 diabetes mellitus. Pediatr Diabetes.

[21] Patel NJ, Savin KL, Kahanda SN, Malow BA, Williams LA, Lochbihler G (2018). Sleep habits in adolescents with type 1 diabetes: Variability in sleep duration linked with glycemic control. Pediatr Diabetes.

[22] Griggs S, Redeker NS, Grey M (2019). Sleep characteristics in young adults with type 1 diabetes. Diabetes Res Clin Pract.

[23] Reutrakul S, Thakkinstian A, Anothaisintawee T, Chontong S, Borel AL, Perfect MM (2016). Sleep characteristics in Type 1 diabetes and associations with glycemic control: systematic review and meta-analysis. Sleep Med.

[24] Chontong S, Saetung S, Reutrakul S (2016). Higher sleep variability is associated with poorer glycaemic control in patients with type 1 diabetes. J Sleep Res.

[25] Hilliard ME, Harris MA, Weissberg-Benchell J (2012). Diabetes resilience: a model of risk and protection in type 1 diabetes. Curr Diab Rep.

[26] Babić R, Babić M, Rastović P, Ćurlin M, Šimić J, Mandić K (2020). Resilience in health and illness. Psychiatr Danub.

[27] Wang RH, Hsu HC, Kao CC, Yang YM, Lee YJ, Shin SJ (2017). Associations of changes in psychosocial factors and their interactions with diabetes distress in patients with type 2 diabetes: a longitudinal study. J Adv Nurs.

[28] Faul F, Erdfelder E, Lang AG, Buchner A (2007). G*Power 3: a flexible statistical power analysis program for the social, behavioral, and biomedical sciences. Behav Res Methods.

[29] Faul F, Erdfelder E, Buchner A, Lang AG (2009). Statistical power analyses using G*Power 31: tests for correlation and regression analyses. Behav Res Methods.

[30] American Psychiatric Association (APA). What is depression? In: Depression. American Psychiatric Association (APA). 2000. https://www.psychiatry.org/patients-families/depression/what-is-depression. Accessed 05 Aug 2022.

[31] Radloff LS (1977). The CES-D scale: a self-report depression scale for research in the general population. Appl Psychol Meas.

[32] Cho MJ, Kim GH (1993). A study on the diagnostic validity of CES-D in the preliminary assessment of patients with major depression. J Korean Neuropsychiatr Assoc.

[33] Kline C. Sleep quality. In: Gellman MD, Turner JR, editors. Encyclopedia of behavioral medicine. New York: Springer; 2013. 10.1007/978-1-4419-1005-9_849.

[34] Buysse DJ, Reynolds CF, Monk TH, Berman SR, Kupfer DJ (1989). The Pittsburgh Sleep Quality Index: a new instrument for psychiatric practice and research. Psychiatry Res.

[35] Choi HJ, Kim SJ, Kim BJ, Kim IJ (2012). Korean versions of self-reported sleep questionnaires for research and practice on sleep disturbance. Korean J Rehabil Nurs.

[36] American Psychological Association. The road to resilience. In: Psychology topics. American Psychological Association. 2014. http://www.apa.org/helpcenter/road-resilience.aspx. Accessed 05 Aug 2022.

[37] Wagnild GM, Young HM (1993). Development and psychometric evaluation of the Resilience Scale. J Nurs Meas.

[38] Jung SH, Park JH, Yoon HJ, Kim JH, Kim NC, Kim SH (2018). The Relationship between sleep quality and depressive symptoms among university students: moderating effect of the positive psychology. J Korean Soc Biol Ther Psychiatry.

[39] Perfect MM, Patel PG, Scott RE, Wheeler MD, Patel C, Griffin K, Sorensen ST, Goodwin JL, Quan SF (2012). Sleep, glucose, and daytime functioning in youth with type 1 diabetes. Sleep.

[40] Wagnild G (2009). The resilience scale user’s guide for the us English version of the resilience scale and the 14-item resilience scale (RS-14).

[41] Neill JT, Dias KL (2001). Adventure education and resilience: the double-edged sword. J Adventure Educ Outdoor Learn.

[42] Liu WJ, Zhou L, Wang XQ, Yang BX, Wang Y, Jiang JF (2019). Mediating role of resilience in relationship between negative life events and depression among Chinese adolescents. Arch Psychiatr Nurs.

[43] Schmitt A, Bendig E, Baumeister H, Hermanns N, Kulzer B (2021). Associations of depression and diabetes distress with self-management behavior and glycemic control. Health Psychol.

[44] Park H (2018). Gender differences in subtypes of depressive symptoms. J Hum Soc Sci.

[45] Song MS, Cho YI (2006). A study of depression, anxiety, stress response, and self-care by gender in diabetic patients. The Korean J Rehabil Nurs.

[46] von Bonsdorff MB, Strandberg A, von Bonsdorff M, Törmäkangas T, Pitkälä KH, Strandberg TE (2017). Working hours and sleep duration in midlife as determinants of health-related quality of life among older businessmen. Age Ageing.

[47] Ji X, Wang Y, Saylor J (2021). Sleep and Type 1 diabetes mellitus management among children, adolescents, and emerging young adults: a systematic review. J Pediatr Nurs.

[48] Smith BW, Dalen J, Wiggins K, Tooley E, Christopher P, Bernard J (2008). The brief resilience scale: assessing the ability to bounce back. Int J Behav Med.

